# How to Tackle the Relationship between Autoimmune Diseases and Diet: Well Begun Is Half-Done

**DOI:** 10.3390/nu13113956

**Published:** 2021-11-05

**Authors:** Camilla Barbero Mazzucca, Davide Raineri, Giuseppe Cappellano, Annalisa Chiocchetti

**Affiliations:** 1 Department of Health Sciences, Interdisciplinary Research Center of Autoimmune Diseases—IRCAD, Università del Piemonte Orientale, 28100 Novara, Italy; camilla.barbero@uniupo.it (C.B.M.); davide.raineri@uniupo.it (D.R.); giuseppe.cappellano@med.uniupo.it (G.C.); 2 Center for Translational Research on Autoimmune and Allergic Disease—CAAD, Università del Piemonte Orientale, 28100 Novara, Italy

**Keywords:** nutrition, dietary assessment, nutritional immunology, Mediterranean diet, western diet, nutritional immunology, autoimmune diseases

## Abstract

Nutrition and immunity are closely related, and the immune system is composed of the most highly energy-consuming cells in the body. Much of the immune system is located within the GI tract, since it must deal with the huge antigenic load introduced with food. Moreover, the incidence of immune-mediated diseases is elevated in Westernized countries, where “transition nutrition” prevails, owing to the shift from traditional dietary patterns towards Westernized patterns. This ecological correlation has fostered increasing attempts to find evidence to support nutritional interventions aimed at managing and reducing the risk of immune-mediated diseases. Recent studies have described the impacts of single nutrients on markers of immune function, but the knowledge currently available is not sufficient to demonstrate the impact of specific dietary patterns on immune-mediated clinical disease endpoints. If nutritional scientists are to conduct quality research, one of many challenges facing them, in studying the complex interactions between the immune system and diet, is to develop improved tools for investigating eating habits in the context of immunomediated diseases.

## 1. Diet and Autoimmune diseases

Autoimmune diseases (ADs) are a family of at least 80 diseases that result from an individual’s immune system attacks the body’s own tissues [1,2,3,4,5]; the pathogenesis and etiology are not fully understood, but environmental factors (lifestyle, diet, drugs, infections) and certain genetic backgrounds have been proposed as risk factors [6]. The incidence of ADs, approximately 3–5% worldwide, is increasing in westernized societies [7], as confirmed by epidemiological studies; these suggest that multiple sclerosis (MS), type 1 diabetes (T1D), inflammatory bowel diseases (mainly Crohn’s disease), systemic lupus erythematosus (SLE), primary biliary cirrhosis, myasthenia gravis, autoimmune thyroiditis, hepatitis, and rheumatic diseases are steadily increasing [8,9]. The geo-epidemiological distribution of ADs, their correlation with socioeconomic status, and their rapid increase in developed countries, together with observations in migrant populations, suggest that environmental factors, rather than genetic ones, are chiefly driving these evolutionary processes [10].

It has been proposed that the increase in ADs throughout Westernized societies over the last three decades could (at least in part) be explained by the increased intestinal permeability induced by industrial food additives [11]. Indeed, in parallel with the observed surge in ADs, industrial food processing and food additive consumption are also expanding [3]. A greater adherence to the Mediterranean diet (MD) in the Mediterranean region has been reported more in elderly people than in younger age groups [12,13]. Changes in agricultural and industrial practices in recent decades have increased food production capacity, through augmented productivity, diversity, and decreased seasonal dependency [14]. These are only some of the phenomena that have created a shift in dietary patterns in Westernized countries. The dietary habits characterized by the consumption of foods high in fats, trans fatty acids, cholesterol, proteins, simple sugars, salt, as well as the frequent consumption of processed and “fast food”, has been termed the “Western diet” (WD). [15,16] In this context, it is hypothesized (and has been demonstrated in vitro) that commonly-used industrial food additives abrogate the human epithelial barrier function; thus, increasing intestinal permeability through the leaky tight junction, resulting in the passage of toxins, food antigens, and bacteria, which may carry immunogenic antigens [3]. Further, depriving the intestinal gut microbiota of dietary fibers, mainly present in fruit, vegetables, legumes, and whole grains, which are not consumed in adequate amounts in Western dietary patterns, could trigger the induction of enzymes capable of degrading the intestinal mucin layer, contributing to the “leaky gut” condition described above [17].

**Figure 1:**
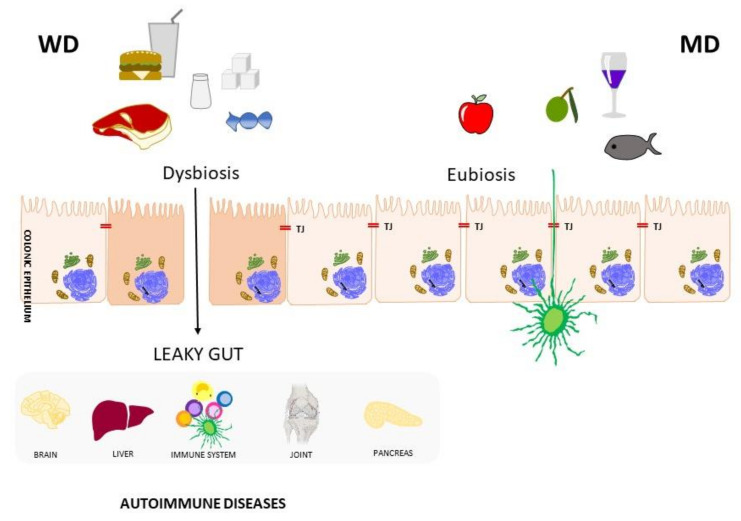
Western diet and Mediterranean diet impacts on gut microbiota homeostasis and intestinal integrity. A leaky gut condition promotes local and systemic inflammation.

Impaired intestinal barrier functions can lead to the activation of an (auto)immune response, which may be induced through different mechanisms, such as molecular mimicry between food ingredients and self-antigens, or the reaction of certain chemical products with self-molecules, generating novel antigenic molecules [3,9]. Furthermore, tight junction dysfunction is a common feature in ADs, and the central role played in AD pathogenesis by altered intestinal permeability has been extensively described [11]. Given these findings, it may be speculated that, in individuals with a genetic predisposition, a leaky gut may trigger the initiation and development of ADs [18] (Figure 1).

In addition to increasing inflammation, the condition of a leaky gut has a second adverse effect: the risk of malabsorption of essential macro- and micronutrients; this is important, since vitamin deficiencies (e.g., vitamin D deficiency) have been named as risk factors for some autoimmune diseases, such as rheumatoid arthritis (RA) and MS [16,19]. In addition to the risk of intestinal barrier damage associated with additives present in ultra-processed foods, the incorrect proportion in the intake of other macronutrients (such as fats), present in large quantities in the WD regimen, can also deregulate the immune system function [20]. The high intake of saturated fats typical of the WD seems to have a direct impact on the innate immune system, by activating toll-like receptors that, in turn, activate pro-inflammatory signaling pathways [21]. In murine models of MS, the effects on the adaptive immune systems observed in animals subjected to high fat diets concerned an increase in the infiltration of T cells and macrophages, associated with a worsening of clinical parameters [22].

Long-chain fatty acids (LCFA), commonly found in processed foods typical of the WD, have been shown to promote differentiation of naïve T cells into pro-inflammatory T helper (TH)-1 and TH17 cells [23]. Moreover, high consumption of refined foods, and excessive calorie intake, induce insulin-resistance and obesity, further increasing the risk of RA [16]. Many correlations have been observed, and many mechanisms proposed, to support the hypothesis that a Westernized food regime may increase the risk of developing autoimmune diseases. Conversely, a protective role of certain diets has also been hypothesized. The literature reports studies on highly restrictive diets (severely calorie-restricted diet), elimination diets, vegetarian diets, the McDougall diet, the Paleolithic diet [23,24], to cite only a few. This review will focus on the MD, since it could be defined as the model opposed to the WD, and since it is the dietary pattern most frequently associated with an improvement in chronic and inflammatory diseases [25].

The Mediterranean diet was first described by Ancel Keys in the early 1960s as the dietary pattern found in the Mediterranean region [26]. It was officially defined in 1993 at the International Conference on the Diets of the Mediterranean, where it was associated with favorable health outcomes [27]. According to its most recent definition, published by UNESCO in 2010, the MD is much more than a diet: rather, it is as a set of cultural traditions and values that interpret food as a means of sharing, of conviviality, aimed at making proper nutrition the pillar of a healthy lifestyle from all standpoints. The MD is characterized by the frequent consumption of olive oil, whole grains, seasonally-available fruits and vegetables, a moderate amount of fish, dairy products, and meat, and many condiments and spices, all accompanied by water, infusions, and wine in moderate amounts [28]. The anti-inflammatory effects of specific nutrients have been demonstrated by studies that clarified the mechanisms underlying the modulating ability of specific nutrients on the immune system [1]. In the context of ADs, polyunsaturated fatty acids (PUFA), found in foods such as fish and oilseeds, have been demonstrated to be able to reduce inflammation when converted into anti-inflammatory prostaglandins (E1, E2), which in turn affect cytokine production and leukocyte migration [29]. Omega-3 fatty acids (eicosapentaenoic acid (EPA) and docosahexaenoic acid (DHA)) are PUFAs involved in regulating inflammatory responses. Conversely, arachidonic acid (AA) and omega-6 fatty acids derived from animal food sources typical of the WD chiefly play pro-inflammatory roles, being precursors of prostaglandins and leukotrienes. However, a recent systematic review highlighted that, overall, omega 3 supplements do not appear to play a significant protective role in preventing one of the most frequent autoimmune diseases, RA. The reason may be that supplementation contributes, only in a small part, to the overall daily intake of fatty acids, meaning that it is not enough to just increase PUFA intake to obtain positive effects [30]. Indeed, rather than the intake levels of omega 3 and omega 6, what appears to be important is their ratio [31]. The optimal omega-6:omega-3 ratio should be 1–4:1, but in the Western diet, it increases to 10–20:1. This imbalance is typical in industrialized countries, and results in an overall increase in inflammation [32]. This suggests the importance of not focusing on individual nutrients to assess their effects on health, but rather considering the overall diet, or at least the overall intake of different food groups.

The high intake of fruits, vegetables, and whole grains, typical of the MD, is primarily beneficial, owing to the high content of fiber, which can support the gut microbiome. Fiber is not an energy source for humans, but it is fermented by bacteria to produce short chain fatty acids (SCFAs), which are able to provide an energy substrate to colonocytes, protecting the intestinal barrier [33]. The ability of microbiota-derived SCFAs in promoting the expansion and differentiation of regulatory T cells (Tregs), which are crucial in maintaining immune homeostasis, has been established, and is a form of chemical-mediated communication between commensal microbiota and the immune system, closely dependent on the diet [34,35]. Tryptophan’s metabolites, which are generated by the diet (e.g., cruciferous vegetables), as well as by gut microbiota, activate the aryl hydrocarbon receptor (AhR), through which the differentiation of FoxP3+ Tregs is induced, and IL-10-producing type 1 regulatory T cells (Tr1). These metabolites are able to cross the blood–brain barrier, reducing local monocyte recruitment and activation, microglial activation, and neurotoxicity, which is important, particularly in MS [23]. A diet rich in fruits and vegetables also provides precious molecules, such as polyphenols, which are secondary plant metabolites, commonly found in tea, coffee, and legumes—characteristic foods of the MD. Epigallocatechin gallate (EGCG), resveratrol, curcumin, and capsaicin possess potent anti-inflammatory properties and are able to modulate signaling pathways that lead to alterations in the expression of pro-inflammatory genes (e.g., cyclooxygenase (COX), lipoxygenase (LOX), phospholipase A2 (PLA2)); these have been studied in depth for their beneficial effects in ADs [30,36].

Therefore, it is evident that there are many bio-plausible explanations regarding the deleterious or protective effects of individual nutrients characteristic of specific nutritional patterns, such as the Mediterranean or Western diets. Unfortunately, it is difficult to correlate them with clinical outcomes, since the sizes of their effects are often small; thus, diets differing in multiple nutrients may be of interest to assess their impact on disease onset [4]. For this reason, the tendency in nutritional research is to focus on food groups and dietary patterns, rather than on individual nutrients [37].

## 2. Starting from the Right Tools

Nutritional interventions have the potential to prevent and/or support the treatment of ADs [27,38]. However, to evaluate the effects that different dietary approaches may have on health, mechanistic studies must be combined with cohort studies to demonstrate the real effects of the diet on the risk of developing autoimmunity. A recent review, aimed at proposing ideas on how to improve the approach of nutritional research, highlighted that understanding the crosstalk between nutrition and immunity requires coordinated interdisciplinary actions, and that the scientific community must make considerable effort toward funding robust, controlled studies. In this context, the use of improved tools to measure dietary intake is a fundamental prerequisite for obtaining quality results [4]. A number of different tools for calculating nutritional intake exist; weighted 24 h (h) recall is the most accurate tool [39] to estimate actual food intake. However, the Food Frequency Questionnaire (FFQ) is often the method of choice used to assess dietary habits in epidemiological studies and trials [40], as it provides the possibility of associating a frequency measure to each food or food category. Further, only those items (if any) considered relevant for the study may be included and standardized data (that are easier to process) are obtained. Conversely, the FFQ is less accurate, and may lead to over/under estimation of dietary intake, which in turn could lead to distorted results. For this reason, it is generally recognized that the FFQ must be validated in the language of use against a standard reference method of dietary assessment, such as a diet record or 24 h recall [41]. Furthermore, it is considered desirable to assess the reproducibility of the instrument by repeatedly administering the questionnaire, to identify variations, and, if possible, assess any “imperfections” in reproducibility caused by measurement errors within the tool or by actual changes in dietary habits between administrations, or both. Therefore, it is clear that the design and validation of FFQs are major experimental tasks. This is confirmed by studies whose sole purposes are to validate questionnaires [42,43], both in terms of internal consistency (i.e., the extent to which the scale items are highly intercorrelated, being neither too homogeneous nor too heterogeneous), and of test–retest reliability (i.e., temporal stability) [44,45].

Given the obvious difficulties in starting from scratch to create a reliable questionnaire, already-validated questionnaires are not infrequently used to carry out nutritional studies. In Italy, for example, most nutritional studies in the field of oncology have been conducted using the questionnaire designed for the (European Prospective Investigation into Cancer and nutrition (EPIC) study [46]. Since this is the only tool available in the Italian language that (relatively precisely) estimates the intake of nutrients, it is combined with the EPIC nutrient database project (ENDB) [47]. It has a matched statistical tool to calculate the intake of macro- and micronutrients, and has been used to assess specific nutrient intakes for diseases other than cancer, including for ADs [48,49]. The EPIC questionnaire is a quantitative food frequency questionnaire, and as such, it consists of a list of foods and drinks divided into categories, for which a frequency of consumption is to be indicated (never, once/several times a week/month/year). Moreover, for some specific items, for example complex carbohydrates (pasta, rice, bread, etc.), animal products (meat, fish, cheese), and some vegetables, the participants must choose among different portion sizes, represented by means of exemplary photographs. Although this is an extremely precise tool for estimating the nutritional intake derived from foods consumed habitually, it is deficient in estimating the consumption of foods typical of the Western diet, such as fast foods; as these are ultra-processed foods, they increase the risk of developing immunomediated diseases. In addition, while investigating the frequency of consumption of meals outside the home, this tool does not provide a detailed indication of the consumption of ready-to-eat foods delivered at home, or at the workplace, through food delivery services, whose frequency of use has increased enormously in recent times https://www.statista.com/study/40457/food-delivery/ accessed on 24 December 2020, not least due to the COVID-19 pandemic. Food prepared away from home has become an important contributor to overall dietary intake, being responsible for 50% of total food expenditure of 2018 in the USA [50]. Thus, it is essential to give importance to this evolution in nutrition, in order to evaluate the effects on health. Indeed, it is now established that food prepared away from home is less healthy than self-prepared food, since it is often energy-dense, high in fat and salt (which have been associated with adverse effects in some autoimmune diseases, such as MS [23] and RA [51]), and deficient in fiber; further, portions are not adapted to individual energy needs [52].

A questionnaire to evaluate adherence to a Westernized dietary regime should also record the origins of the products consumed, since the spread of supermarkets is a fundamental “protagonist” in the nutritional transition that has taken place in industrialized countries; this is also associated with the increase of non-communicable diseases [53] https://www.theguardian.com/food/2020/feb/13/how-ultra-processed-food-took-over-your-shopping-basket-brazil-carlos-monteiro accessed on 13 February 2020. EPIC FFQ investigates the places where fruits and vegetables are usually purchased. However, it is also important to evaluate the preference for processed products, such as “sandwich bread”, easily available in the supermarkets, over fresh baked goods, or for animal products derived from large intensive farms, which are the most common suppliers of supermarkets, over local products that can be purchased in the butcher’s shop [14]. The fact that the questionnaire drawn up for the EPIC study does not include these aspects is understandable, since it dates back to the 1990s; therefore, the need for a more up-to-date questionnaire is becoming increasingly apparent. Conversely, it would be of considerable interest to evaluate not only individual nutrients, but also general dietary patterns (important because of the closer associations with outcomes), and any synergistic effects of specific foods on the health outcomes investigated (a possibility, demonstrated by a famous analysis from the Nurses’ Health Study [54] and by recent studies in the field of nutritional immunology [4,14,37]). To this end, it would be useful to design a new tool that not only contains questions aimed at determining adherence to the WD, but that also only keeps items that help to determine adherence to a traditional/Mediterranean diet. Such a new tool, for investigating eating habits, would fill unmet needs of nutritional immunologists, who want to understand and study the relationship between diet and the immune system. These two opposing diets could represent important risk and prevention factors, respectively, which have the potential to change the way in which we practice primary and secondary prevention of immune-mediated diseases.

## 3. Dietary Patterns and Evaluation Methods

**Figure 2:**
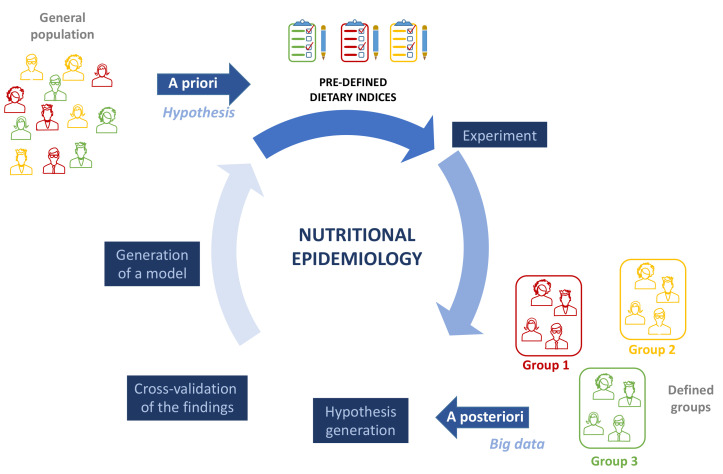
Two main approaches may be used to study the role of dietary patterns as a modifiable lifestyle factor in autoimmune diseases. The a priori or hypothesis-driven approach examines the adherence to pre-defined dietary indices in relation to AD development or disease relapse. On the contrary, a posteriori or data-driven approaches use statistical tools—dimension reduction methods or clustering—to investigate the relationships between ADs and diet. Given that a posteriori approach evaluates big data, analysis may generate a new hypothesis, which, once validated, may establish new models for a priori testing.

There are two main ways of approaching dietary pattern analyses: “a priori” or hypothesis driven and “a posteriori” or data driven (Figure 2). In the “a priori” evaluation, nutritional variables that are important for health, based on scientific evidence and on recommendations provided by nutritional or dietetics institutions, or other models (such as the United States Department of Agriculture’s food guide pyramid, the Mediterranean pyramid, the UK’s Eatwell guide), are quantified and summed to create a score/index that gives a comprehensive measure of the quality of the diet. On the contrary, “a posteriori” method uses statistical tools (factor analysis, PCA, cluster analysis) to derive dietary patterns from data collected from a study population. Numerous published studies have applied this method to attempt to derive significantly different food patterns in different subgroups of the sample being studied, so that they can be associated with variations in biomarkers or specific outcomes [55,56,57]. There are a lack of scientific data comparing the two aforementioned approaches to dietary pattern analysis [58]. However, for the goal of carrying out studies aimed at providing evidence-based notions to be used for preventive interventions at the population level, the “a priori” method, where different studies suggest the beneficial or harmful effects of foods belonging to well-defined patterns, seems to be more functional. The “a posteriori” method may not be generalized to the overall population; indeed, the aforementioned tools used to collect dietary information for this method are somewhat subjective and may vary by socioeconomic status, ethnic groups, and cultures, so that they may not be generalizable to the reference population [56,59]. Noteworthy, a posteriori approach, by evaluating big data, may generate a new hypothesis, which once validated, may establish new models for a priori testing (Figure 2).

Both methods proved to be extremely useful in estimating the associations between diet and specific immune/pathological conditions, as well as particular age groups [60,61]. Some adherence scores have been coined to evaluate associations with other diseases or on specific population groups. These include the PREDIMED study, which led to the development of the Mediterranean Diet of Screener Adherence (MEDAS) score, which includes 14 questions on the main food groups of the MD; a higher MEDAS score is positively correlated with high-density lipoprotein cholesterol (HDL), and negatively correlated with body mass index, waist circumference, triglycerides (TG), and fasting glucose. However, the results cannot be generalized to the general population, since the participants were elderly individuals at high risk of coronary hearth diseases [43]. The KIDMED questionnaire, published in 2004 in *Public Health Nutrition*, was the first specifically designed to evaluate adherence to MD in children and adolescents, through a 16-item Mediterranean Diet Quality Index; it has been used for more than a decade by nutritional scientists [62].

Scores determined from mean population intakes derived from meta-analyses [63] based on different cohort studies, or according to dietary guidelines [64], are preferred when applying to the general population [65]. In regard to a cohort of 22,043 Greek adults, Trichopoulou and colleagues demonstrated that, in their population-based prospective study, adherence to the MD, calculated for the first time with a score built on the salient characteristics of this diet, was positively associated with a lower total mortality and cancer and cardiovascular disease-related mortality [66]. Several studies have also been performed on the EPIC cohort, in which adherence to the MD was evaluated by means of other tools, such as the adapted relative MD (arMED) score and the MedDiet pyramid (pyramid) score, to study the effects on different (mainly oncologic and non-communicable) diseases [67,68,69]. Another (more recent) tool, the Medi-Lite questionnaire (the name derives from the two words “Mediterranean” and “Scientific Literature”), was created by Francesco Sofi, Associate Professor of Food Sciences at the University of Florence, who aimed to develop a practical and easily-used tool to evaluate adherence to the Mediterranean diet, based on scientific data, concerning its beneficial effects. It comprises a small number of questions about nine food groups, which enable adherence to the MD to be evaluated. It is freely available, to increase knowledge among the general population about the importance of correct nutrition [70]. Moreover, given the wide variety of proposed MD scores, some studies [28] have attempted to provide a defined nutrient content and range of servings for the MD. These are based on the available literature and aim to provide a more “universal” definition, by calculating an average quantity of foods and nutrients from previous scoring systems. They seek to match traditional and recent relevant evidence and provide a reference profile of the MD, which may be used to design intervention MD comparable to other studies. To the present authors’ knowledge, however, no adherence score to the MD has been developed specifically to assess its effects on immune-mediated diseases.

## 4. Many MD Scores, Few WD Scores

In general, it should be noted that the great majority of nutritional studies only evaluate “one side of the coin”, i.e., they only consider the “goodness” of the diet (see the above-mentioned studies), without considering the “inappropriateness”. This means that the evaluation is not complete, and precludes an overall evaluation of the food patterns followed by the subjects in the study. Fewer studies evaluate adherence to the WD, and those few are mainly based on empirically derived “a posteriori” methods [55]. Some studies evaluate adherence to the WD versus adherence to a ‘prudent’ diet, often seen as antithetical to the Westernized regimen (being characterized by more frequent intakes of vegetables, fruits, cooking/dressing oil, cereals and legumes, whole grains, rice/pasta, fish, low-fat dairy, poultry, and water), using “a posteriori” methods, namely factor analysis, or factor analysis together with the Alternate Healthy Eating Index (AHEI), an “a priori” scoring method constructed based on expert opinions, representing optimal dietary behavior for disease prevention [36,71]. To date, similar studies that evaluate adherence to the WD vs. MD, in a combined way with “a priori” methods, are lacking, particularly in the context of nutritional immunology.

## 5. Need for a Double Score

**Figure 3:**
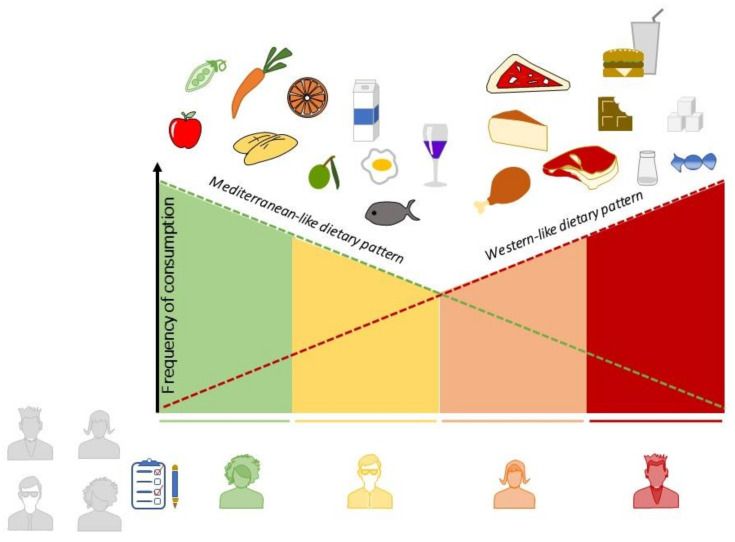
A MD–WD double adherence score to obtain a comprehensive, overall vision of dietary habits.

Given the large number of studies indicating the possible role of the WD as a risk factor for immunomediated diseases, there is a need for an instrument capable of theoretically deriving the adherence to this diet, which is now as precisely defined in terms of characteristic foods [15,20,25], as is the MD. It would be of considerable use if, for subjects participating in a nutritional study, their positions could be determined upon a “scale” of adherence running from the MD, at one end, to the WD, at the other. The Western dietary pattern is now predominant in industrialized countries [72], partly thanks to factors inherent in contemporary society that do not depend on the will of individuals, such as globalization, and the consequent evolution of food production techniques. Thus, it would be of great interest to evaluate how closely a subject “approaches” the traditional dietary pattern, while perhaps living in a globally Westernized context. Moreover, a double score could be more precise than a single MD score, since it would permit researchers to assign “negative” values to excessive consumption of certain foods, such as fruits, which should not exceed 3/4 portions per day. Excessive fruit consumption enhances the risk of exceeding the amount of simple sugars ingested, which should be limited in the MD [73] (Figure 3).

## 6. Concluding Remarks

Nutritional immunology is a complex field of study, given the lack of uniformity of a “diet” as an input factor. To date, studies indicating the protective or harmful effects of specific nutrients and foods are not lacking, and the mechanisms by which what we eat being able to impact our immune function have also been clarified. Despite the scientific utility of these notions, the effect-size of individual nutrients/foods is limited when considered within the context of a complex dietary pattern, in which possible synergies between foods also come into play. It is therefore necessary to focus on nutritional patterns and study them, employing large cohorts that are representative of the general population, if one is to obtain evidence-based indications to be used for the implementation of preventive interventions on the population. Concerning two food patterns, both of which have been studied in depth for their effects, respectively, pro- and anti-inflammatory, namely the MD and WD, numerous prerequisites must be considered, in terms of their possible harmful/preventive effects modifying the risk of developing immunomediated diseases. However, to date, no study has been able to demonstrate these effects. One reason for this gap is the lack of a tool that, starting from the degree of adherence to the two patterns considered, enables the true association with the risk of developing the disease to be clarified. Indeed, to study such an association, a single score, able to evaluate adherence to one of the two patterns, for example the MD, may not suffice. Rather, we need a double tool, which gives an idea of the degree of appropriateness of the diet, and which can be used on different populations with different backgrounds. In the USA, a diet that is a little more “Mediterranean” than the common WD regime might correspond to a diet at the limit of “Mediterraneity” in Italy, where, until a few decades ago, the MD was a traditional pattern. For this reason, the diet of each individual should be positioned on an ideal scale that goes from what is believed to be the most harmful to the most protective pattern. The creation of a food questionnaire, from which it is possible to estimate a score of “Westernity” or “Mediterraneity” could constitute a universal tool that is able to provide a comprehensive, overall vision of the dietary habits of study subjects, providing a solid instrumental basis upon which to conduct high-quality studies. Although, until now, little importance has been given to the tools used to conduct nutritional studies, and since questionnaires designed for other diseases or scores not suitable for the study population are currently being used, nutritional immunologists should think about instruments capable of evaluating such complex exposures. Beginning with the right approach will make it easier to obtain solid evidence with which to demonstrate that nutrition plays a fundamental role in preventing inflammatory and immune-mediated diseases, and to promote the “food for health” culture, which will benefit the population in terms of public health and environmental impact.

## Data Availability

Not applicable.

## References

[1] Çehreli R. (2018). Moleculer nutritional immunology and cancer. J. Oncol. Sci..

[2] Platt A.M. (2017). Immunity in the Gut. Viral Gastroenteritis.

[3] Lerner A., Matthias T. (2015). Changes in intestinal tight junction permeability associated with industrial food additives explain the rising incidence of autoimmune disease. Autoimmun. Rev..

[4] Venter C., Eyerich S., Sarin T., Klatt K.C. (2020). Nutrition and the immune system: A complicated tango. Nutrients.

[5] Rose N.R. (2016). Prediction and prevention of autoimmune disease in the 21st century: A review and preview. Am. J. Epidemiol..

[6] De Luca F., Shoenfeld Y. (2019). The microbiome in autoimmune diseases. Clin. Exp. Immunol..

[7] Okada H., Kuhn C., Feillet H., Bach J.-F. (2010). The “hygiene hypothesis” for autoimmune and allergic diseases: An update. Clin. Exp. Immunol..

[8] Selmi C. (2010). The worldwide gradient of autoimmune conditions. Autoimmun. Rev..

[9] Brady B.D.M. (2014). Autoimmune disease: A modern epidemic? Molecular mimicry, the hygiene hypothesis, stealth infections, and other examples of disconnect between medical research and the practice of clinical medicine. N. Engl. J. Med..

[10] Fatoye F., Gebrye T., Svenson L.W. (2018). Real-world incidence and prevalence of systemic lupus erythematosus in Alberta, Canada. Rheumatol. Int..

[11] Fasano A., Shea-Donohue T. (2005). Mechanisms of disease: The role of intestinal barrier function in the pathogenesis of gastrointestinal autoimmune diseases. Nat. Clin. Pract. Gastroenterol. Hepatol..

[12] Buckland G., Agudo A., Travier N., Huerta J.M., Cirera L., Tormo M.-J., Navarro C., Chirlaque M.D., Moreno-Iribas C., Ardanaz E. (2011). Adherence to the Mediterranean diet reduces mortality in the Spanish cohort of the European Prospective Investigation into Cancer and Nutrition (EPIC-Spain). Br. J. Nutr..

[13] Naska A., Trichopoulou A. (2014). Back to the future: The Mediterranean diet paradigm. Nutr. Metab. Cardiovasc. Dis..

[14] Monteiro C.A., Cannon G., Levy R.B., Moubarac J.C., Louzada M.L.C., Rauber F., Khandpur N., Cediel G., Neri D., Martinez-Steele E. (2019). Ultra-processed foods: What they are and how to identify them. Public Health Nutr..

[15] Varlamov O. (2017). Western-style diet, sex steroids and metabolism. Biochim. Biophys. Acta -Mol. Basis Dis..

[16] Gioia C., Lucchino B., Tarsitano M.G., Iannuccelli C., Di Franco M. (2020). Dietary habits and nutrition in rheumatoid arthritis: Can diet influence disease development and clinical manifestations?. Nutrients.

[17] Desai M.S., Seekatz A.M., Koropatkin N.M., Kamada N., Hickey C.A., Wolter M., Pudlo N.A., Kitamoto S., Terrapon N., Muller A. (2016). A dietary fiber-deprived gut microbiota degrades the colonic mucus barrier and enhances pathogen susceptibility. Cell.

[18] Maione F., Cappellano G., Bellan M., Raineri D., Chiocchetti A. (2020). Chicken-or-egg question: Which came first, extracellular vesicles or autoimmune diseases?. J. Leukoc. Biol..

[19] Munger K.L., Levin L.I., Hollis B.W., Howard N.S., Ascherio A. (2006). Serum 25-hydroxyvitamin D levels and risk of multiple sclerosis. JAMA.

[20] Christ A., Lauterbach M., Latz E. (2019). Western diet and the immune system: An inflammatory connection. Immunity.

[21] Huang S., Rutkowsky J.M., Snodgrass R.G., Ono-Moore K.D., Schneider D.A., Newman J.W., Adams S.H., Hwang D.H. (2012). Saturated fatty acids activate TLR-mediated proinflammatory signaling pathways. J. Lipid Res..

[22] Timmermans S., Bogie J.F.J., Vanmierlo T., Lütjohann D., Stinissen P., Hellings N., Hendriks J.J.A. (2014). High fat diet exacerbates neuroinflammation in an animal model of multiple sclerosis by activation of the renin angiotensin system. J. Neuroimmune Pharmacol..

[23] Katz Sand I. (2018). The role of diet in multiple sclerosis: Mechanistic connections and current evidence. Curr. Nutr. Rep..

[24] Hagen K.B., Byfuglien M.G., Falzon L., Olsen S.U., Smedslund G. (2009). Dietary interventions for rheumatoid arthritis. Cochrane Database Syst. Rev..

[25] Cena H., Calder P.C. (2020). Defining a healthy diet: Evidence for the role of contemporary dietary patterns in health and disease. Nutrients.

[26] Martínez-González M.A., Sánchez-Villegas A. (2004). The emerging role of Mediterranean diets in cardiovascular epidemiology: Monounsaturated fats, olive oil, red wine or the whole pattern?. Eur. J. Epidemiol..

[27] Forsyth C., Kouvari M., D’Cunha N.M., Georgousopoulou E.N., Panagiotakos D.B., Mellor D.D., Kellett J., Naumovski N. (2018). The effects of the Mediterranean diet on rheumatoid arthritis prevention and treatment: A systematic review of human prospective studies. Rheumatol. Int..

[28] Davis C., Bryan J., Hodgson J., Murphy K. (2015). Definition of the mediterranean diet: A literature review. Nutrients.

[29] Calder P.C. (2017). Omega-3 fatty acids and inflammatory processes: From molecules to man. Biochem. Soc. Trans..

[30] Nelson J., Sjöblom H., Gjertsson I., Ulven S.M., Lindqvist H.M., Bärebring L. (2020). Do interventions with diet or dietary supplements reduce the disease activity score in rheumatoid arthritis? A systematic review of randomized controlled trials. Nutrients.

[31] Simopoulos A.P. (2008). The importance of the omega-6/omega-3 fatty acid ratio in cardiovascular disease and other chronic diseases. Exp. Biol. Med..

[32] Anderson B.M., Ma D.W.L. (2009). Are all n-3 polyunsaturated fatty acids created equal?. Lipids Health Dis..

[33] Statovci D., Aguilera M., MacSharry J., Melgar S. (2017). The impact of western diet and nutrients on the microbiota and immune response at mucosal interfaces. Front. Immunol..

[34] Atarashi K., Tanoue T., Oshima K., Suda W., Nagano Y., Nishikawa H., Fukuda S., Saito T., Narushima S., Hase K. (2013). Treg induction by a rationally selected mixture of Clostridia strains from the human microbiota. Nature.

[35] Arpaia N., Campbell C., Fan X., Dikiy S., van der Veeken J., de Roos P., Liu H., Cross J.R., Pfeffer K., Coffer P.J. (2013). Metabolites produced by commensal bacteria promote peripheral regulatory T-cell generation. Nature.

[36] Strate L.L., Keeley B.R., Cao Y., Wu K., Giovannucci E.L., Chan A.T. (2017). Western dietary pattern increases, and prudent dietary pattern decreases, risk of incident diverticulitis in a prospective cohort study. Gastroenterology.

[37] Bennett E., Peters S.A.E., Woodward M. (2018). Sex differences in macronutrient intake and adherence to dietary recommendations: Findings from the UK Biobank. BMJ Open.

[38] Zaccardelli A., Friedlander H.M., Ford J.A., Sparks J.A. (2019). Potential of lifestyle changes for reducing the risk of developing rheumatoid arthritis: Is an ounce of prevention worth a pound of cure?. Clin. Ther..

[39] Sharma M., Rao M., Jacob S., Jacob C.K. (1998). Validation of 24-hour dietary recall: A study in hemodialysis patients. J. Ren. Nutr..

[40] Steinemann N., Grize L., Ziesemer K., Kauf P., Probst-Hensch N., Brombach C. (2017). Relative validation of a food frequency questionnaire to estimate food intake in an adult population. Food Nutr. Res..

[41] Fatihah F., Ng B.K., Hazwanie H., Karim Norimah A., Shanita S.N., Ruzita A.T., Poh B.K. (2015). Development and validation of a food frequency questionnaire for dietary intake assessment among multi-ethnic primary school-aged children. Singap. Med. J..

[42] Affret A., El Fatouhi D., Dow C., Correia E., Boutron-Ruault M.C., Fagherazzi G. (2018). Relative validity and reproducibility of a new 44-item diet and food frequency questionnaire among adults: Online assessment. J. Med. Internet Res..

[43] García-Conesa M.T., Philippou E., Pafilas C., Massaro M., Quarta S., Andrade V., Jorge R., Chervenkov M., Ivanova T., Dimitrova D. (2020). Exploring the validity of the 14-item mediterranean diet adherence screener (Medas): A cross-national study in seven european countries around the mediterranean region. Nutrients.

[44] Turconi G., Celsa M., Rezzani C., Biino G., Sartirana M.A., Roggi C. (2003). Reliability of a dietary questionnaire on food habits, eating behaviour and nutritional knowledge of adolescents. Eur. J. Clin. Nutr..

[45] Goldbohm R.A., Van’t Veer P., Van den Brandt P.A., Van’t Hof M.A., Brants H.A.M., Sturmans F., Hermus R.J.J. (1995). Reproducibility of a food frequency questionnaire and stability of dietary habits determined from five annually repeated measurements. Eur. J. Clin. Nutr..

[46] Riboli E., Kaaks R. (1997). The EPIC project: Rationale and study design. European Prospective Investigation into Cancer and Nutrition. Int. J. Epidemiol..

[47] Slimani N., Deharveng G., Unwin I., Southgate D.A.T., Vignat J., Skeie G., Salvini S., Parpinel M., Møller A., Ireland J. (2007). The EPIC nutrient database project (ENDB): A first attempt to standardize nutrient databases across the 10 European countries participating in the EPIC study. Eur. J. Clin. Nutr..

[48] de Pablo P., Romaguera D., Fisk H.L., Calder P.C., Quirke A.-M., Cartwright A.J., Panico S., Mattiello A., Gavrila D., Navarro C. (2018). High erythrocyte levels of the n-6 polyunsaturated fatty acid linoleic acid are associated with lower risk of subsequent rheumatoid arthritis in a southern European nested case—control study. Ann. Rheum. Dis..

[49] Vagnani S., Tani C., Carli L., Querci F., Della Rossa A., D’Ascanio A., Ermini I., Ceroti M., Caini S., Palli D. (2014). Nutritional assessment in patients with systemic lupus erythematosus and systemic sclerosis. Arthritis Rheumatol..

[50] Keeble M., Adams J., Sacks G., Vanderlee L., White C.M., Hammond D., Burgoine T. (2020). Use of online food delivery services to order food prepared away-from-home and associated sociodemographic characteristics: A cross-sectional, multi-country analysis. Int. J. Environ. Res. Public Health.

[51] Salgado E., Bes-Rastrollo M., de Irala J., Carmona L., Gómez-Reino J.J. (2015). High sodium intake is associated with self-reported rheumatoid arthritis: A cross sectional and case control analysis within the SUN cohort. Medicine.

[52] McGuire S., Todd J.E., Mancino L., Lin B., Jessica E. (2011). The impact of food away from home on adult diet quality. USDA-ERS Econ. Res. Rep. Pap. Adv. Nutr..

[53] Demmler K.M., Klasen S., Nzuma J.M., Qaim M. (2017). Supermarket purchase contributes to nutrition-related non-communicable diseases in urban Kenya. PLoS ONE.

[54] Heidemann C., Schulze M.B., Franco O.H., van Dam R.M., Mantzoros C.S., Hu F.B. (2008). Dietary patterns and risk of mortality from cardiovascular disease, cancer, and all causes in a prospective cohort of women. Circulation.

[55] Moore L.V., Diez Roux A.V., Nettleton J.A., Jacobs D.R., Franco M. (2009). Fast-food consumption, diet quality, and neighborhood exposure to fast food: The multi-ethnic study of atherosclerosis. Am. J. Epidemiol..

[56] Ramezankhani A., Hosseini-Esfahani F., Mirmiran P., Azizi F., Hadaegh F. (2021). The association of priori and posteriori dietary patterns with the risk of incident hypertension: Tehran Lipid and Glucose Study. J. Transl. Med..

[57] Newby P.K., Tucker K.L. (2004). Empirically derived eating patterns using factor or cluster analysis: A review. Nutr. Rev..

[58] Hu F.B., Rimm E., Smith-Warner S.A., Feskanich D., Stampfer M.J., Ascherio A., Sampson L., Willett W.C. (1999). Reproducibility and validity of dietary patterns assessed with a food-frequency questionnaire. Am. J. Clin. Nutr..

[59] Panagiotakos D.B., Pitsavos C., Stefanadis C. (2009). α-priori and α-posterior dietary pattern analyses have similar estimating and discriminating ability in predicting 5-y incidence of cardiovascular disease: Methodological issues in nutrition assessment. J. Food Sci..

[60] Leermakers E.T.M., van den Hooven E.H., Franco O.H., Jaddoe V.W.V., Moll H.A., Kiefte-de Jong J.C., Voortman T. (2018). A priori and a posteriori derived dietary patterns in infancy and cardiometabolic health in childhood: The role of body composition. Clin. Nutr..

[61] Khaled K., Hundley V., Almilaji O., Koeppen M., Tsofliou F. (2020). A priori and a posteriori dietary patterns in women of childbearing age in the UK. Nutrients.

[62] Serra-Majem L., Ribas L., Ngo J., Ortega R.M., García A., Pérez-Rodrigo C., Aranceta J. (2004). Food, youth and the Mediterranean diet in Spain. Development of KIDMED, Mediterranean Diet Quality Index in children and adolescents. Public Health Nutr..

[63] Sofi F., Macchi C., Abbate R., Gensini G.F., Casini A. (2013). Mediterranean diet and health status: An updated meta-analysis and a proposal for a literature-based adherence score. Public Health Nutr..

[64] Monteagudo C., Mariscal-Arcas M., Rivas A., Lorenzo-Tovar M.L., Tur J.A., Olea-Serrano F. (2015). Proposal of a Mediterranean diet serving score. PLoS ONE.

[65] Hodge A., Bassett J. (2016). What can we learn from dietary pattern analysis?. Public Health Nutr..

[66] Trichopoulou A., Costacou T., Bamia C., Trichopoulos D. (2003). Adherence to a Mediterranean diet and survival in a Greek population. N. Engl. J. Med..

[67] Solans M., Benavente Y., Saez M., Agudo A., Naudin S., Hosnijeh F.S., Noh H., Freisling H., Ferrari P., Besson C. (2019). Adherence to the Mediterranean diet and lymphoma risk in the European prospective investigation into cancer and nutrition. Int. J. Cancer.

[68] Shannon O.M., Stephan B.C.M., Granic A., Lentjes M., Hayat S., Mulligan A., Brayne C., Khaw K.-T., Bundy R., Aldred S. (2019). Mediterranean diet adherence and cognitive function in older UK adults: The European Prospective Investigation into Cancer and Nutrition-Norfolk (EPIC-Norfolk). Study. Am. J. Clin. Nutr..

[69] Trichopoulou A., Orfanos P., Norat T., Bueno-de-Mesquita B., Ocké M.C., Peeters P.H.M., van der Schouw Y.T., Boeing H., Hoffmann K., Boffetta P. (2005). Modified Mediterranean diet and survival: EPIC-elderly prospective cohort study. BMJ.

[70] Sofi F., Dinu M., Pagliai G., Marcucci R., Casini A. (2017). Validation of a literature-based adherence score to Mediterranean diet: The MEDI-LITE score. Int. J. Food Sci. Nutr..

[71] Shakersain B., Santoni G., Larsson S.C., Faxén-Irving G., Fastbom J., Fratiglioni L., Xu W. (2016). Prudent diet may attenuate the adverse effects of Western diet on cognitive decline. Alzheimer’s Dement..

[72] Cunnane S.C. (2005). Origins and evolution of the Western diet: Implications of iodine and seafood intakes for the human brain. Am. J. Clin. Nutr..

[73] Bach-Faig A., Berry E.M., Lairon D., Reguant J., Trichopoulou A., Dernini S., Medina F.X., Battino M., Belahsen R., Miranda G. (2011). Mediterranean diet pyramid today. Science and cultural updates. Public Health Nutr..

